# Spatially Controlled, Wirefree, Drug Release along a Single PEDOT Film

**DOI:** 10.1021/acs.langmuir.6c00755

**Published:** 2026-05-14

**Authors:** Áine Brady, Ewa Bieniasz, Robert Forster

**Affiliations:** † School of Chemical Sciences, 8818 Dublin City University, Dublin 9 D09 V209, Ireland; ‡ FutureNeuro, The Research Ireland Centre for Translational Brain Science, Dublin City University, Dublin 9 D09 V209, Ireland

## Abstract

A wire-free bipolar electrochemical strategy for spatially resolved release of encapsulated molecules from within a conducting polymer film is described. Thin electrodeposited PEDOT films were loaded with fluorescein and exposed to an externally generated electric field (∼0.6 V cm^–1^), creating a longitudinal potential gradient within the PEDOT film. The resulting spatial variation in PEDOT oxidation state modulates fluorescein release, producing programmable, region-dependent release along the single continuous film. This enables directional drug elution (low-to-high release across the film) without the need for wires, embedded electronics, or segmented reservoirs. This, wirefree, spatially nonuniform drug release enables applications where the drug needs to be delivered preferentially to one tissue, *e.g.*, to reduce irritation or toxicity to adjacent healthy tissue, *e.g.*, ocular inserts, periodontal chips, wound dressings, stents, orthopedic implants *etc.*, where a drug release is only beneficial on one area of the device. Moreover, reconfiguring the external field distribution provides a route to translate the release zone over time enabling dynamic targeting postdeployment.

## Graphical abstract

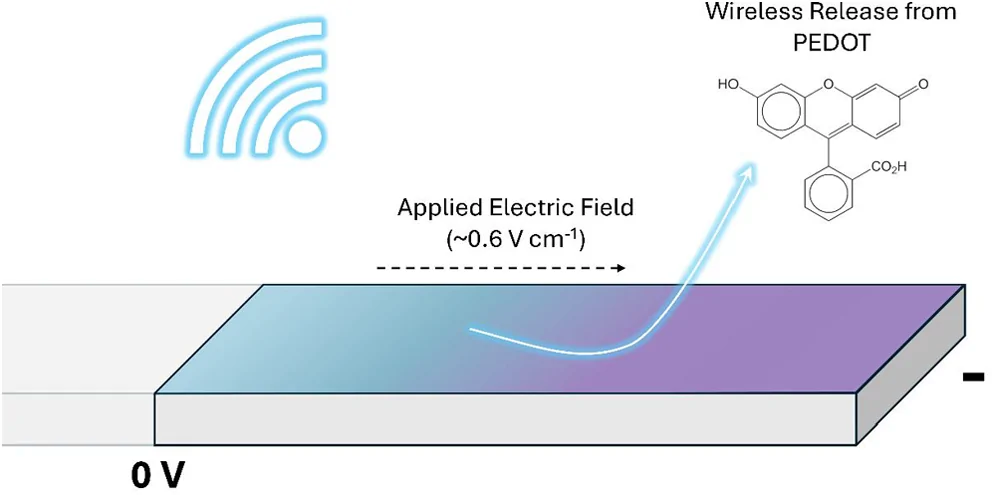

## Introduction

Most drug-eluting coatings release their payload in an intrinsically spatial uniform despite the need for controlled concentration gradients, *i.e.*, high at the target tissue and minimal at adjacent healthy tissue to limit off-target exposure. Moreover, there is a strong need for “on-demand” systems where drug release is part of a closed loop control system where drug is only released in response to an analytical measurement, *e.g.*, an increased concentration of a disease biomarker. Bipolar or “Wirefree” electrochemistry is especially attractive in this context, since a bipolar electrode (BPE) does not need to be physically connected to an instrument to drive redox reactions, *e.g.*, to switch the oxidation state of a conducting polymer to drive molecular release.^1^ The approach uses two or more feeder electrodes to generate a gradient electric field in solution that induces a potential on the remote BPE.^2,3^ Recently, the distribution of the wirelessly generated electric fields has been mapped using conducting polymers and electrochemiluminescent dyes to understand the magnitude of the induced voltages for biological and environmental applications.^4–6^ Wirefree electrochemistry has also gained attention in the areas of multiplexed devices, ultrasensitive biosensors in conjunction with electrochemiluminescence, electroceuticals, and wireless drug delivery.^7–11^

Conducting polymers such as poly­(3,4-ethylenedioxythiophene), PEDOT, are attractive materials due to their stability, flexibility, and superior conductivity.^12^ Their redox switching capabilities, surface charge, and conductivity provide them the potential to act as drug delivery vehicles for the controlled and targeted delivery of Active Pharmaceutical Ingredients (APIs),^13^ electrostimulation of nerve cells,^14^ and the potential to be used as materials for cell destruction, such as the wireless stimulation of bacteria.^15^ Here, PEDOT has been used which is a *p*-type doping polymer, where oxidation creates positive charges along the polymer backbone causing negatively charged species in the supporting electrolyte to ingress, or if reduced, driving the release of anions.^16^ When used in conjunction with wireless electrochemistry, wirefree drug delivery systems (WDDS) hold promise as therapeutic platforms for chronic conditions, having the ability to perform precise and controlled delivery. Additionally, these on-demand platforms could be designed with dual purpose such as the simultaneous stimulation of cells and delivery of drugs for localized cancer cell apoptosis, utilizing the conductivity, morphology and biocompatibility of polymer films for cell adhesion and thus, stimulation and drug delivery. Furthermore, the ability to perform *in situ* simultaneous polymerization and API doping on conducting medical implantable devices (*e.g.*, hip replacements, coronary stents) paves way for continuous controlled drug release with the ability for recharging *in situ*. Similarly, implantable closed-loop drug delivery systems (DDS) often require external wiring (*e.g.*, insulin pump for diabetics). These restrictions further highlight the necessity for smart delivery devices that maximize precision and control, while minimizing user burden for patients.

Here, we present the use of bipolar electrochemistry together with the conducting polymer PEDOT for the wireless delivery of the model drug fluorescein. Fluorescein is a common model anionic drug in the medical industry since its fluorescence can be used to conveniently track release and quantify payload. This contribution investigates the impact of gradient dedoping *via* wireless electric fields and electrostimulation time on the efficiency of wireless fluorescein dedoping from PEDOT films in electrolyte. The results provide promising insights into the stability, morphology, homogeneity, film thickness, drug loading capabilities and wireless dedoping efficiency of the PEDOT films. These insights enable a method by which therapeutics can be controlled and released at specific tumor sites in a minimally invasive method, having the potential to reduce adverse side effects, drug toxicity, and most importantly, increase drug efficacy and patient outcomes. The ability to localize redox reactions without the need of physical wiring represents a significant advancement in the development of WDDS, in particular the administration of cytotoxic drugs such as chemotherapeutics.

## Experimental Section

### Materials

All materials used throughout this experimentation were of reagent grade, obtained from Merck, and used as received. Milli-Q water used throughout this experimentation had a resistivity of 18.2 MΩ cm.

### Conventional Three-Electrode Cell

#### Electrochemical Characterization of Fluorescein

Cyclic voltammetry was performed using a CHI660 potentiostat. A glassy carbon electrode (GCE) was used as the working electrode, Ag/AgCl (saturated KCl) reference electrode, and a coiled platinum wire counter electrode (≈3.6 cm^2^). Fluorescein studies were performed in 10 mM fluorescein sodium salt in 0.1 M LiClO_4_. Film capacitance was calculated from the slope of *i*_pa_ *versus* scan rate. Film thickness was calculated by assuming a volumetric capacitance of 100 F cm^–3^ for the PEDOT/ClO_4_ film.^17^

#### Electrodeposition of PEDOT/ClO_4_ Films

Electropolymerization was performed in a three-electrode cell using chronocoulometry with an initial voltage of 1.1 V in 10 mM EDOT in 0.1 M LiClO_4_. 2 cm × 1 cm Fluorine-doped Tin Oxide (FTO) glass was taped with Teflon tape to give a working area of 1 cm × 1 cm. Electrodeposition was performed until 40 mC charge passed. Films were rinsed with Milli-Q water and ClO_4_^–^ dedoping was performed in 0.1 M LiClO_4_.

#### Conventional Doping

Conventional doping of fluorescein into the PEDOT film was performed using chronoamperometry by applying a constant voltage of +0.5 V for 300 s. Doping was performed in a 10 mM fluorescein sodium salt solution in Milli-Q water.

#### Conventional Dedoping

Conventional dedoping was performed using chronoamperometry, applying a constant −0.5 V to reduce PEDOT for 600 s. Dedoping was performed in a known sample volume of 0.1 M LiClO_4_ (6 mL).

#### Wireless Dedoping

Wireless dedoping was performed in a bipolar electrochemical cell that was designed in-house with FreeCAD and printed using the Ultimaker S5 3D printer in tough black PLA (Figure 3(a)). The cell consists of two, *L*:2 cm × *W*:1 cm × *D*:2 mm titanium feeder electrodes, with a separation (channel length) of 3 cm. The feeder electrodes were polished using 1200 grit sandpaper (≈12 μm particle size) between experiments. A *L*:2 cm ± 0.2 cm × *W*:1 cm ± 0.2 cm FTO on glass (2 mm) electrode was located central to the feeder electrodes. The FTO BPEs were sonicated in alcohol and dried under nitrogen prior to use. The FreeCAD stl file was exported for compatibility with Cura software, where parameters such as resolution, infill density, and temperature were optimized for printing. The cell was printed using “normal” resolution (0.15 mm), with an infill density of 20% at 230 °C. The theoretical voltage induced across the BPE was calculated using the following EF strength equation^2^

1 $$\Delta E_{\mathrm{elec}}=E_{\mathrm{tot}}\left(\frac{l_{\mathrm{elec}}}{l_{\mathrm{channel}}}\right)$$

where *l*_channel_ is the distance between driving electrodes (constant at 3 cm), *l*_elec_ is the length of the BPE, and *E*_tot_ is the voltage difference applied to the pair of feeders. In the following description, the negative feeder is considered to be on the left-hand side so that the negative pole of the BPE, where dedoping takes place, lies on the right.

#### Scanning Electron Microscopy

A Jeol JSM-IT 100 InTouchScope electron microscope was used to image the PEDOT films on FTO upon polymerization and after wireless dedoping.

#### Wide-Field Fluorescence Microscopy

The Echo Revolution Upright/Inverted Fluorescence Microscope was used to image PEDOT/ClO_4_ and PEDOT/FLSN films on FTO. The fluorescein isothiocyanate (FITC) channel was used for excitation (450 to 490 nm) and emission wavelengths (500 to 550 nm).

## Results and Discussion

### Electrochemical Characteristics of Fluorescein

Fluorescein (FLSN) is a xanthene synthetic organic dye that is well-known for its superior fluorescence and photostability.^18^ It has a characteristic green emission at approximately 510 nm when excited at 370 nm, making it a useful tool in biological and physicochemical studies.^19^ Fluorescein sodium salt was chosen as the “model” drug to represent the doping and dedoping capabilities of the conducting polymer film because of its p*K*_a_ of 2.2, 4.4, and 6.7,^20^ meaning the compound remains in its dianionic form at pH 7. This ensures that the compound remains negatively charged for optimal doping and dedoping of the *p*-type doping polymer PEDOT, which exhibits a positively charged backbone upon oxidation. Additionally, fluorescence spectroscopy is an ultrasensitive analytical technique, allowing for the accurate quantification of drug loading and release (370 nm) (Figure S1). Understanding the electrochemical behavior of the dopant is essential to creating stable, homogeneous films, especially under the conditions required for release. It has been previously reported that fluorescein exhibits a strong oxidation peak on the initial voltametric scan, followed by a flat and featureless response on sequential scans, demonstrating the irreversible behavior of fluorescein oxidation (Figure S2).^21^ This behavior suggests that a nonelectroactive film is formed through the oxidation of the fluorophore, which blocks the electrode surface, preventing electropolymerization of the monomer EDOT and therefore inhibiting a PEDOT/FLSN film from being formed, at least for this setup.

### Electrochemical Characterization of PEDOT Films

The conducting polymer PEDOT is widely employed in bioelectronic applications due to its high conductivity, stability, and biocompatibility. PEDOT films are electrochemically synthesized through the oxidation of the EDOT monomer which involves the removal of a ϖ-electron and therefore the formation of cationic radicals. To achieve electroneutrality, anions move into the positively charged polymer.^22,23^ Since PEDOT films cannot be formed in the presence of fluorescein, the PEDOT film was first electrodeposited using chronocoulometry in a 10 mM EDOT 0.1 M LiClO_4_ solution at +1.1 V. To ensure film reproducibility, film formation was stopped when 40 mC charge passed, since there is a direct correlation with charge passed and film thickness (Figure 1(a)).

**Figure 1:**
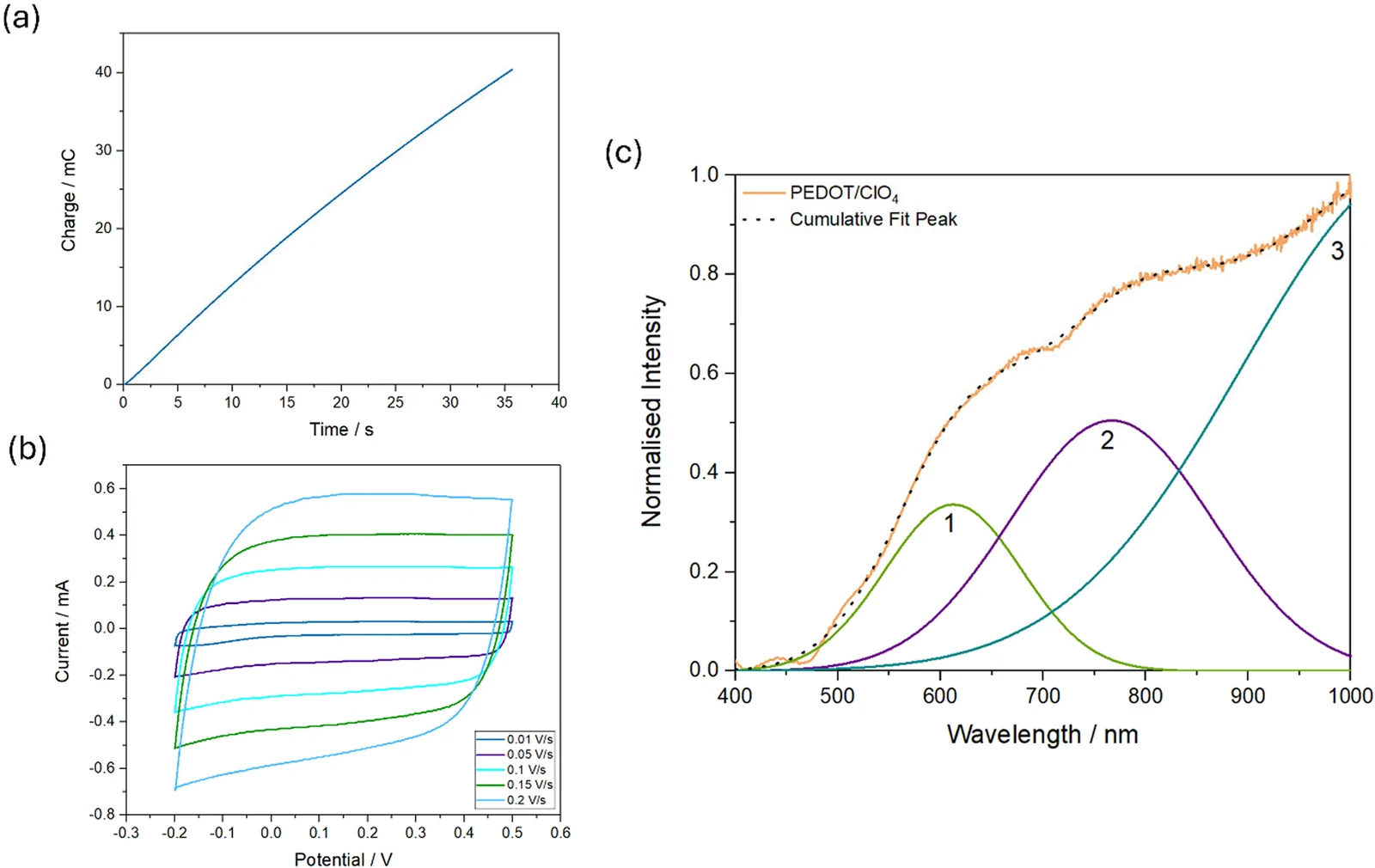
(a) Chronocoulometric response for EDOT polymerization in 10 mM EDOT 0.1 M LiClO_4_ in Milli-Q water at +1.1 V. (b) Scan rate study of the 40 mC PEDOT/ClO_4_ film cycled in 0.1 M LiClO_4_ (10–200 mV s^–1^). (c) Normalized UV–vis spectrum from 400 to 1000 nm of a 40 mC PEDOT/ClO_4_ film on FTO glass (blank FTO glass). The curves 1, 2, and 3 represent the Gaussian deconvolution that best represents the spectrum (black dotted line).

Cyclic voltammetry was used to quantify the capacitance of the 40 mC PEDOT/ClO_4_ film measured from the current using a series of scan rate studies from 10 to 200 mVs^–1^, as illustrated in Figure 1(b). The film capacitance was calculated at 0.2 V, where the current is governed by nonfaradaic processes. The film capacitance depends on the quantity of PEDOT deposited and can provide information on film thickness and physicochemical properties. Film thickness was calculated by assuming a volumetric capacitance of 100 F cm^–3^ for the PEDOT/ClO_4_ film,^17^ corresponding to a film thickness of 285 nm (2.61 × 10^–7^ moles).

### UV–Vis Spectroscopy

The level of doping of the PEDOT films are of particular interest to encourage optimal loading as well as having sufficient conductivity to perform dedoping experiments. Factors such as polymerization method (*i.e.*, galvanostatic, potentiometric), doping ions, applied voltages, and deposition rates can affect the structure, composition, and doping states of the film. Electronically conducting polymers gain their conductivity through doping, with high doping polymers reaching levels of 5000 S/cm and low/undoped films having levels of 0.1 S/cm.^24,25^ Figure 1(c) features the 40 mC PEDOT/ClO_4_ film UV–vis spectrum showcasing strong absorbance from 600 to 1000 nm. Gaussian deconvolution was used to resolve the wavelengths of the individual electronic transitions identified as peaks 1, 2, and 3. The first peak observed at approximately 600 nm can be attributed to a polaronic electronic transition with the second polaron-related band occurring at 750 nm, both of which are characteristic of partially oxidized PEDOT, reflecting the presence of charge carriers introduced during doping.^26^ At 1000 nm, peak 3 arises from higher energy polaron transitions, with the possibility of bipolaron absorption.^26^ The UV spectrum reflects a substantial level of oxidative doping, offering good capacity for uptake of negatively charged molecules through electrostatic doping.

### Microscopy

SEM is a powerful technique that is used to understand the structure and morphology of polymer films. Here, SEM was used to determine the homogeneity of the PEDOT films on FTO after conventional polymerization by chronocoulometry. Figure 2(a) shows the layered film on the FTO substrate after polymerization in 10 mM EDOT in 0.1 M LiClO_4_. The light surface texturing of the film could potentially provide cell adhesion, which would further broaden the use of this substrate for dual applications such as simultaneous drug release and cell stimulation.^14^ To assess morphological changes associated with wireless FLSN release on the PEDOT film, the surface of the BPE was also analyzed using SEM Figure 2(b) showcases the cathodic side of the BPE (Right Hand Side (RHS)) after wireless FLSN release. A notable change in film texture is visible, revealing a less homogeneous film coverage. Toward the edge of the BPE, small amounts of film delamination can be seen, likely due to the application of negative voltages.

**Figure 2:**
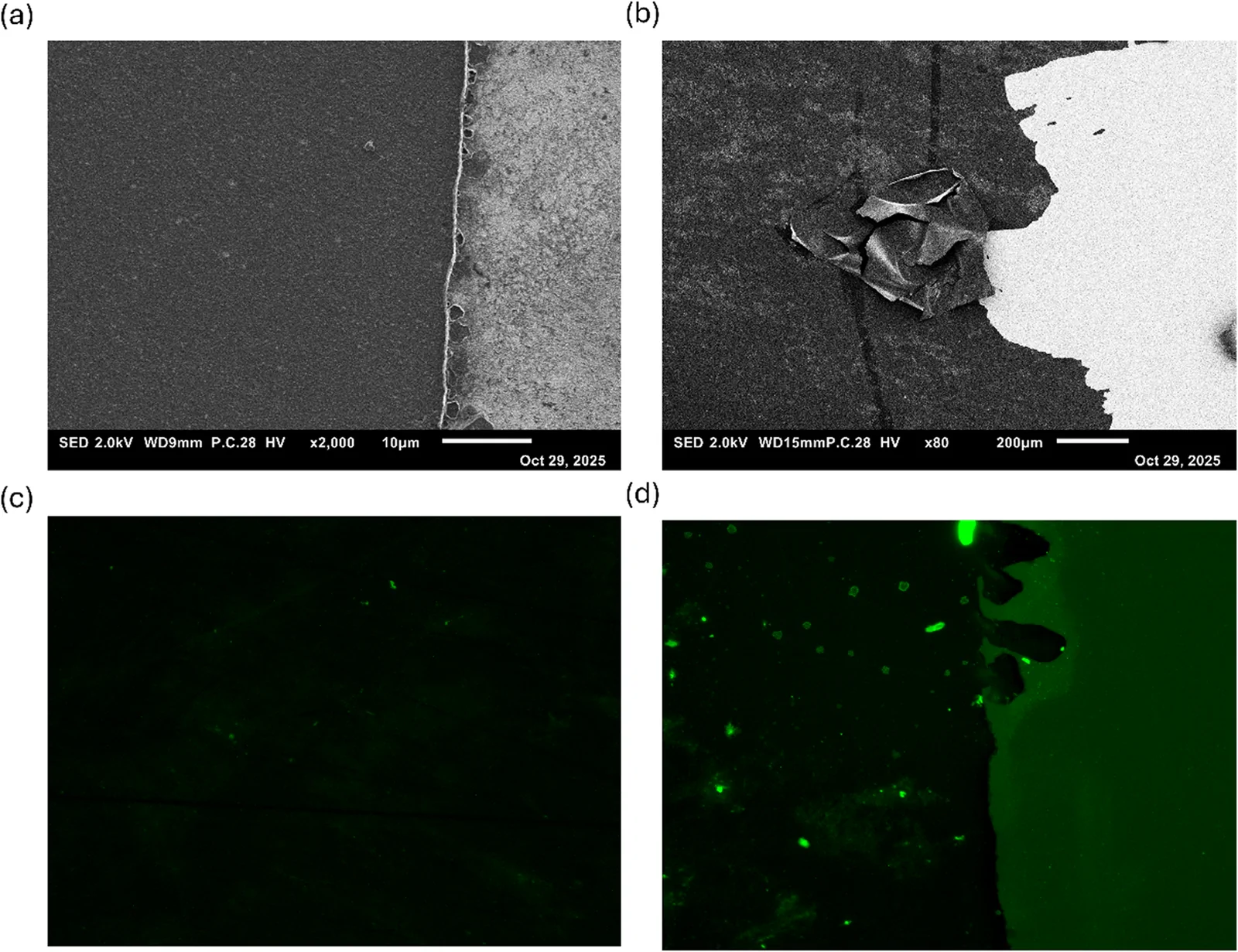
SEM image of the PEDOT film (a) directly after polymerization in 10 mM EDOT 0.1 M LiClO_4_ (PEDOT film located to the left in the image) and (b) after wireless dedoping of fluorescein at 10 V (0.62 V cm^–1^)^27^ for 10 min in 0.1 M LiClO_4_ (PEDOT film located to the left in the image). Wide-field fluorescence microscopy of (c) PEDOT/ClO_4_ film directly after polymerization as described above (PEDOT film is the entire image) and (d) after conventional doping in 10 mM fluorescein sodium salt for 5 min (PEDOT film located to the left in the image).

Wide-field fluorescence microscopy enables fluorescent species to be visualized in real time through optical excitation and emission. The intensity of the illumination of the fluorophore does not correspond directly with the concentration at that point, due to (i) polymer quenching of the fluorophore,^28^ and (ii) changes to the extinction coefficient due to the environment (polymer entrapment).^29^ Here, fluorescence microscopy was used to demonstrate the distribution of fluorescein in the doped PEDOT/FLSN film, using a PEDOT/ClO_4_ film as a control.

Figure 2(c) shows the PEDOT/ClO_4_ film is essentially nonfluorescent, as evidenced by the uniformly dark background. Only some small voids within the film are faintly outlined by a weak green halo which is most likely attributed to excitation light scattering since the Stokes shift is small.^30^ Figure 2(d) displays the bright green emission characteristic of fluorescein distributed throughout the film. It should be noted that the underlying FTO substrate also appears green, which again arises from light refraction within the glass itself rather than any intrinsic fluorescent material in the substrate.

### Conventional Drug Loading and Release

Chronoamperometry was employed to perform drug loading and release of fluorescein from the PEDOT films (1 cm × 1 cm on FTO). Through the application of a constant potential, the resulting current–time response provided insights into the ion exchange kinetics and the efficiency of the redox driven transport within the polymer matrix. Before the fluorescein loading could take place, dedoping of perchlorate ions was performed by exhaustively reducing the PEDOT film at −0.5 V for 10 min. Drug loading was performed by oxidizing the film at +0.5 V for 300 s in 10 mM fluorescein sodium salt in the absence of deliberately added electrolyte. The oxidation current decreases over time as a result of capacitive charging and limitations in counterion diffusion into the film due to the film being fully oxidized (Figure S3­(a)). Once doped with fluorescein, the dye could be released by reducing the PEDOT film at −0.5 V for 600 s which is illustrated in Figure S3­(b) by the increase in current over this time frame. Current increase in this scenario is attributed to the loss of anions from the polymer film, however, it is important to note that as reduction continues, positively charged ions can also be incorporated into the film for charge neutrality purposes.^12^

To evaluate the release performance of both the conventional and wireless systems, a calibration curve of fluorescein sodium salt solutions ranging from 30 to 300 nM (*n* = 3) was created (Figure S1­(b)). Based on this, conventional three-electrode chronoamperometry released (1.12 ± 0.10) × 10^–9^ moles of fluorescein from the 285 nm PEDOT film (2.61 × 10^–7^ moles of EDOT) (*n* = 2).

### Wireless Dedoping of Fluorescein

#### Influence of Voltage of PEDOT Dedoping

Bipolar electrochemistry was employed to perform the wireless dedoping of the conventionally doped PEDOT/FLSN films with two 2 cm × 1 cm × 2 mm titanium foil feeder electrodes (Figure 3(a)). The BPE (2 cm × 1 cm FTO) was positioned so that the conducting polymer was at the negative side of the BPE, facing the positive feeder electrode (Figure 3(b)). The effects of voltage on the wireless dedoping of fluorescein from the PEDOT film was investigated by applying external voltages of 5, 10, and 15 V (power supply voltages) at a constant time of 10 min. These voltages were based on predicted voltages from previous work despite the differences in electrolyte concentration,^27^ where edge voltages were predicted to prevent over reducing the film and inhibiting its electrical properties. At low power supply voltages of 5 V (0.31 V cm^–1^), the potential distribution across the negative side of the BPE (polymer film), is sufficiently negatively charged to release a small quantity of fluorescein as seen in Figure 3(c); however, this voltage does not maximize the entire polymer film as voltages toward the center of the BPE, 0 V, are possibly too low to release more of the model drug *i.e.*, the film remains in the “semidoped” state.

**Figure 3:**
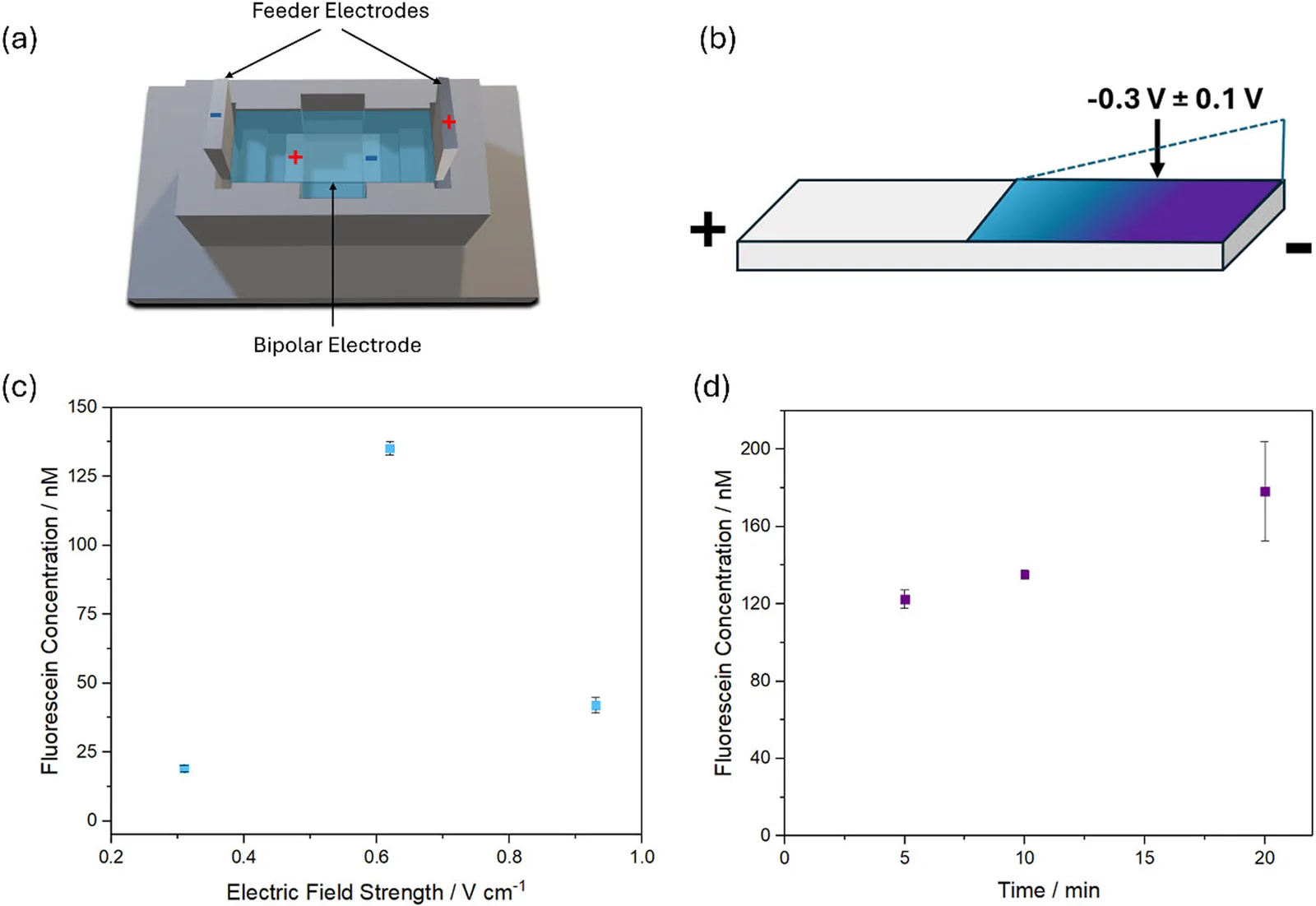
(a) CAD design of the BP setup with two titanium feeder electrodes separated by a 3 cm solution channel. (b) Diagram of the PEDOT/FLSN film after wireless dedoping where the polymer color change predicts the electrode voltage at that position. (c) Electric field strength study on the wireless release of fluorescein from the PEDOT film in 0.1 M LiClO_4_ for 10 min. (d) Effect of time on the wireless release of fluorescein from the PEDOT film in 0.1 M LiClO_4_ at 10 V.

At the high induced potential of 15 V (0.93 V cm^–1^), the quantity of drug released dramatically reduces to just above the 5 V quantity. This is due to the higher field strength causing over reduction of the polymer film which results in processes like film degradation, swelling and delamination, all of which would limit the redox properties of the PEDOT film.^31,32^ A power supply voltage of 10 V (0.62 V cm^–1^) was deemed the optimal doping potential as a 3-fold increase in material was released from the conducting polymer film (135 nM, (4.05 ± 0.21) × 10^–10^ moles).

To examine the wireless release of fluorescein from the PEDOT film, dedoping was performed for 5, 10, and 15 min. The aim of this study was to determine the stability of the film at negative voltages, while simultaneously assessing the influence of dedoping time on drug release. The effect of time on drug release, Figure 3(d), shows that most of the fluorescein was released within the first 5 min of dedoping, which correlates with the rapid release chronoamperometry profiles indicating that drug release is primarily controlled by the redox kinetics of the conducting polymer, rather than by prolonged application of the field. The film dedoped wirelessly at 10 V for 10 min released approximately half the quantity ((4.05 ± 0.21) × 10^–10^ moles) of the conventional release at −0.5 V for 10 min ((1.12 ± 0.10) × 10^–9^ moles), demonstrating the effects of the gradient wireless potential on the BPE, in comparison to the constant potential applied to the conventional platform.

Figure 4 displays the electric field strengths of PEDOT systems using two methods (i) electric field equation (eq 1), and (ii) polymer reduction potential. PEDOT exhibits a color change when transitioning from oxidized to reduced states, with fully doped PEDOT films having a light to navy blue coloring and transitioning to a purple color when in the dedoped state. ImageJ software was used to map redox induced color changes in PEDOT, where the area over which the film changes from oxidized (blue) to reduced (purple) was calculated to be 0.514 cm^2^ on the wirelessly released film. To predict the voltage at the color transition, PEDOT/FLSN films were conventionally dedoped at 0.0 V, −0.2 V and −0.4 V, concluding that the color change occurs at approximately −0.3 V ± 0.1 V (Figure S4), which corresponds with UV analysis in literature.^33,34^ Based on the location of the band (Figure S4) and assuming an onset reduction voltage of −0.3 V ± 0.1 V, an electric field strength for the 10 V system was calculated at 0.62 V cm^–1^ significantly lower than the 3.33 V cm^–1^ predicted by eq 1 and from previous work,^27^ however, this is likely due to the significant increase in electrolyte concentration.

**Figure 4:**
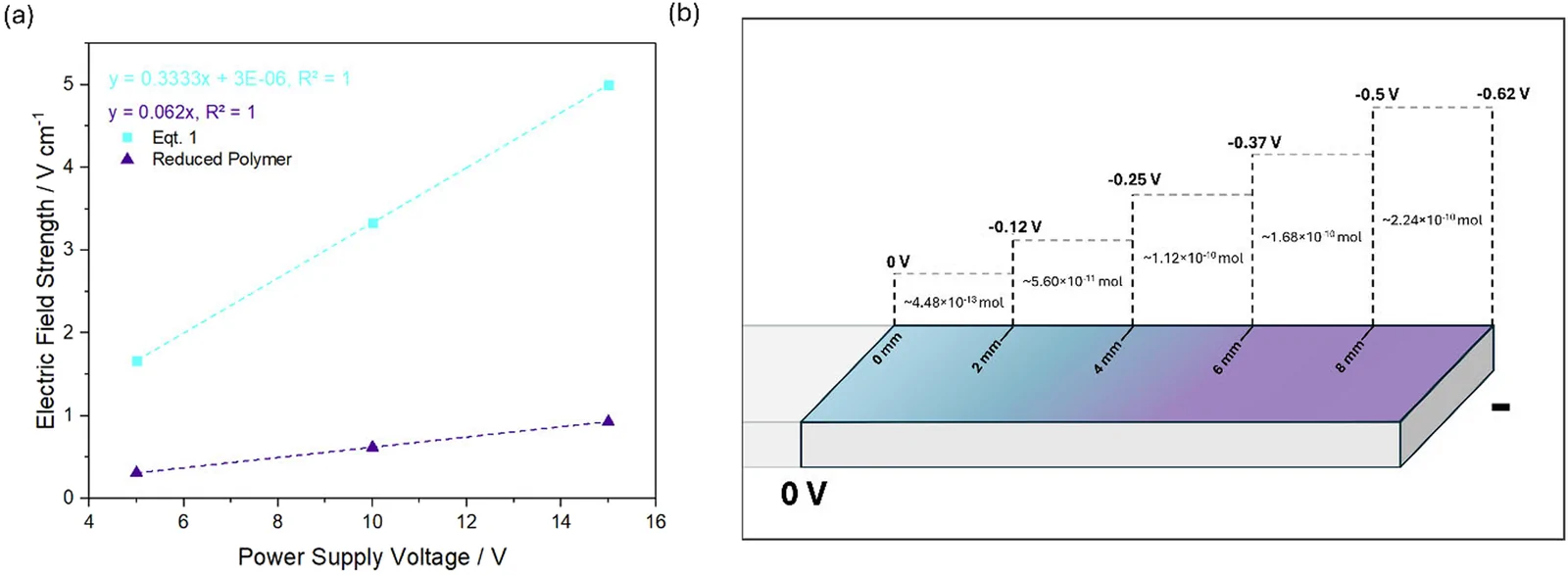
(a) The calculated electric field strengths based on the electric field equation (eq 1) (blue), and the initiation of polymer reduction depicted by color change and analyzed using ImageJ software assuming reduction occurs at −0.3 ± 0.1 V (purple). (b) Theoretical estimation of the spatial release of fluorescein across the varying BPE voltages.

Assuming that the redox behavior of the PEDOT/FLSN film follows Nernstian behavior, such that it responds predictably to changes in electrochemical potential, the amount of fluorescein released along the length of the BPE, and thus at different local potentials, was predicted based on conventional dedoping at −0.5 V, as shown in Figure 4(b). Using this approach, the spatial release was estimated, yielding a predicted total release of 5.60 × 10^–10^ moles, which is in good agreement with the experimentally measured value of 4.05 × 10^–10^ moles. By varying the externally applied voltage, the potential distribution along the BPE is modulated accordingly, enabling spatially resolved control of fluorescein release along its length. This capability provides a means for achieving spatially controlled release, with potential application in targeted delivery of APIs, however, it is important to note that APIs of varying molecular weights will have varying doping capabilities.

## Conclusion

This study demonstrates that thin-film PEDOT platforms can support electrochemically controlled therapeutic loading and wireless, spatially resolved release, while also offering insight into electric field behavior in bipolar electrochemical systems. Spectroscopic analysis confirms that PEDOT/ClO_4_ films are dominated by polaronic charge carriers, facilitating ion exchanging during doping and dedoping. Importantly, wireless electrical stimulation was shown to reliably induce the release of encapsulated fluorescein from ultrathin PEDOT films (285 nm). By optically mapping release across the polymer surface, an electric field strength of ∼0.62 V cm^–1^ was calculated for the system, creating a potential gradient within the PEDOT film. Together these observations provide important considerations for the design and translation of wireless drug delivery and sensing platforms intended for operation in highly conductive biological environments.

## Supplementary Material



## Notes

Chemical structure for fluorescein and fluoresence calibration curve of fluroescein sodium salt; cyclic voltammetry of fluorescein forming a non-electroactive compound upon oxidation; and real photographs of PEDOT films dedoped conventionally and wirelessly (PDF)
