# Supplementary material for: Spatially Controlled, Wirefree, Drug Release along a Single PEDOT Film
Source: Langmuir. 2026 May 14;42(21):14827–34. doi: 10.1021/acs.langmuir.6c00755 (PMC13637076; doi:10.1021/acs.langmuir.6c00755)
Supplement: Supplementary file 1 [file la6c00755_si_001.pdf]

Supporting Information

Spatially Controlled, Wirefree, Drug Release Along a Single PEDOT Film

Áine Brady[a,b*], Ewa Bieniasz,[a] Robert Forster [a,b*]

[a] School of Chemical Sciences,

Dublin City University, Dublin 9, Ireland, D09 V209

[b]FutureNeuro, The Research Ireland Centre for Translational Brain Science, Dublin City University.

Corresponding Authors: Prof. Robert Forster, School of Chemical Sciences,

Dublin City University, Dublin 9, Ireland, D09 V209. **Email:** robert.forster@dcu.ie

Dr. Áine Brady, School of Chemical Sciences,

Dublin City University, Dublin 9, Ireland, D09 V209. **Email:** aine.brady@dcu.ie

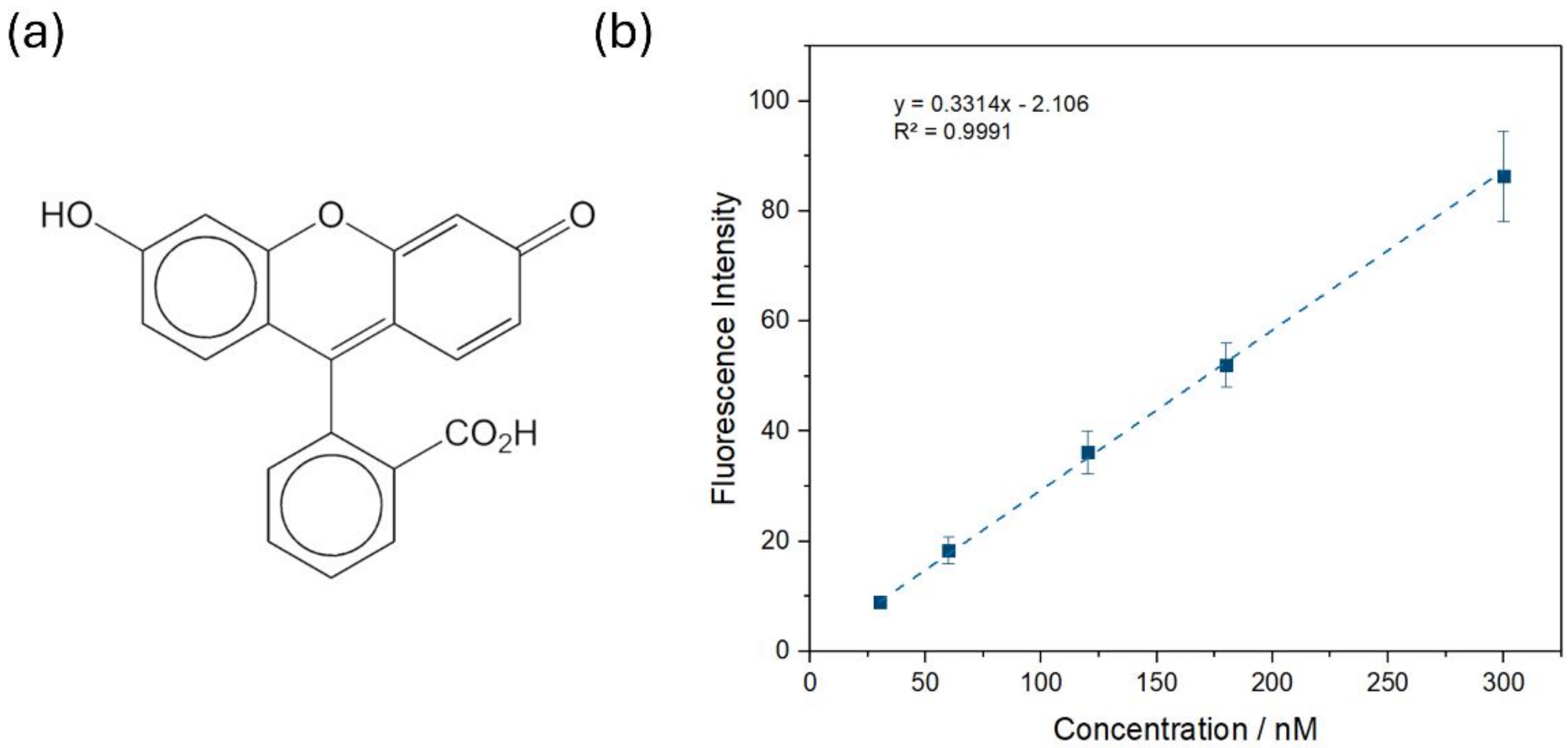

Figure S1. (a) Chemical structure of fluorescein (b) Fluorescence intensity calibration curve of fluorescein sodium salt in Milli-Q water ranging from 30 to 300 nM (n=3).

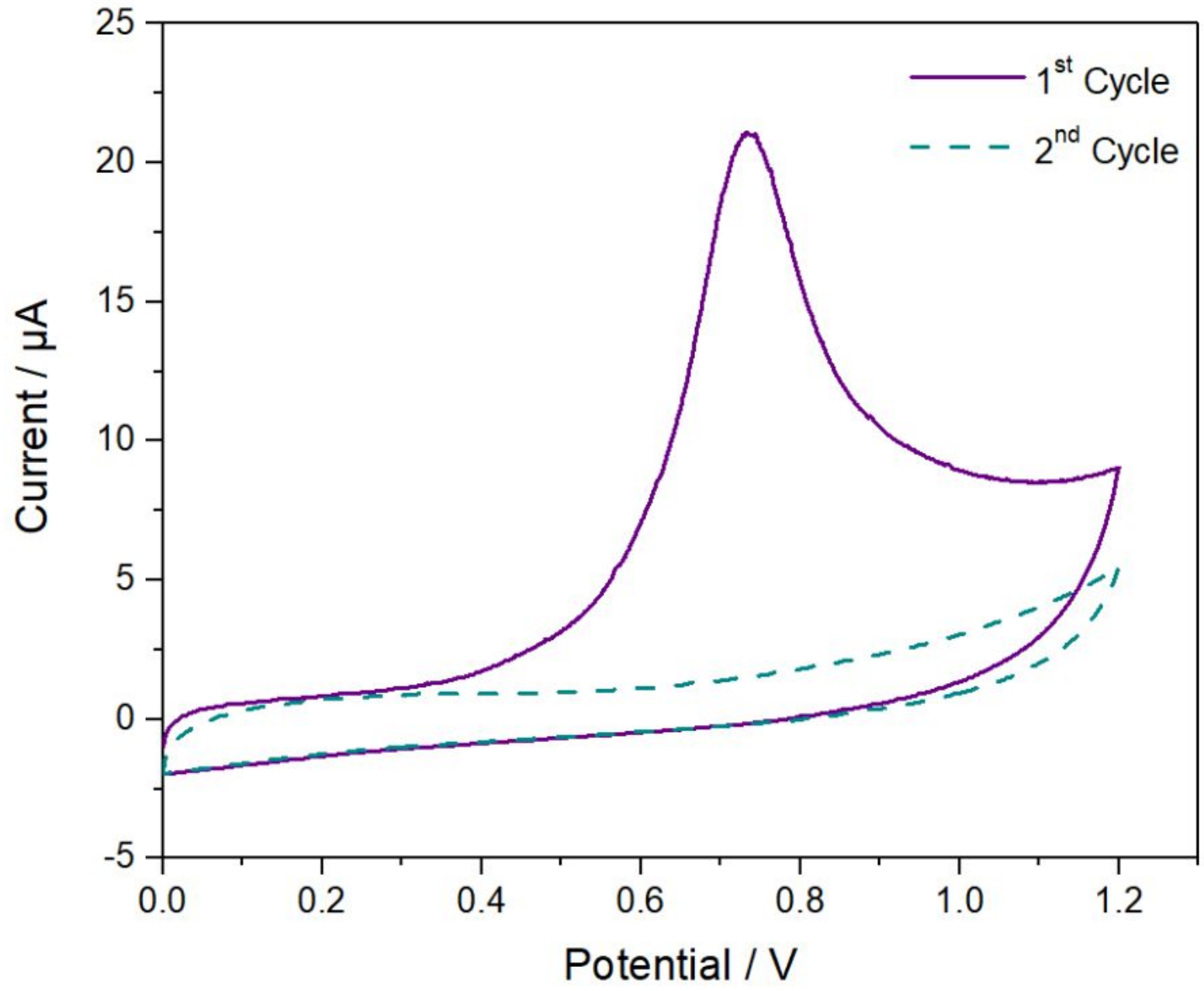

Figure S2. Cyclic voltammogram of 10 mM Fluorescein sodium salt in 0.1 M $LiClO_4$ on a glassy carbon electrode.

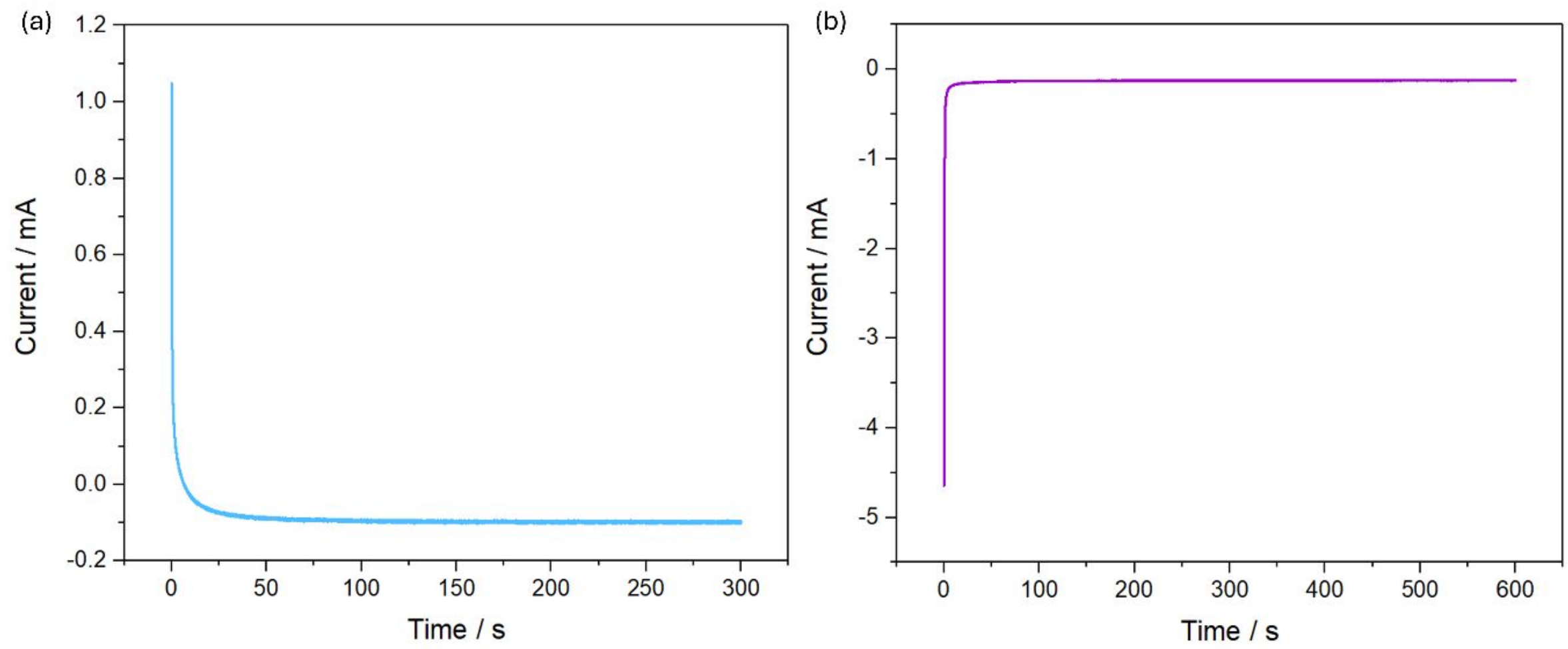

Figure S3. Current versus time responses for the conventional (a) loading of 10 mM fluorescein for 5 minutes at +0.5 V and (b) release of fluorescein for 10 minutes at -0.5 V in 0.1 M $LiClO_4$. Experiments were performed on 40 mC PEDOT films.

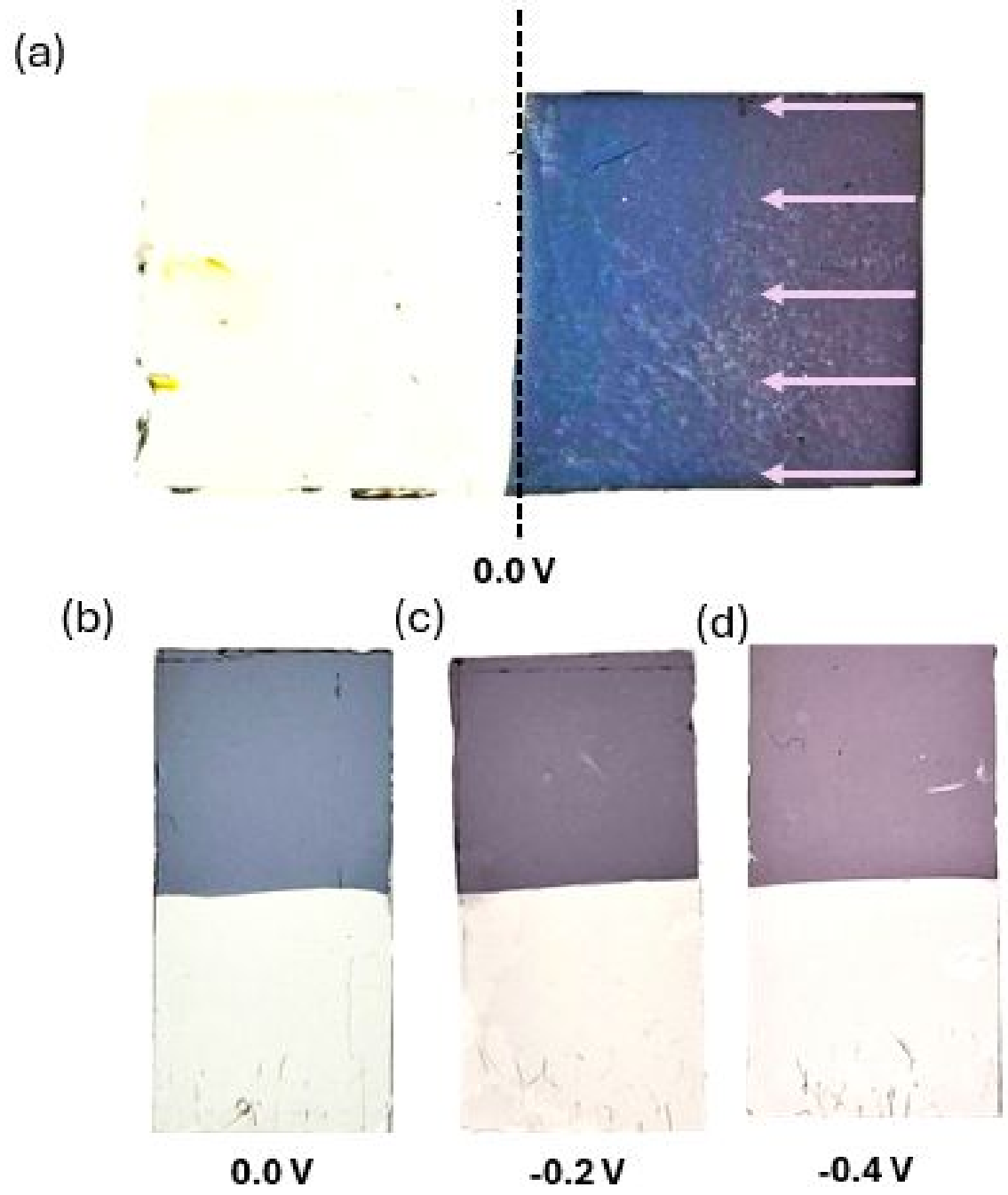

Figure S4. Photograph of 40 mC PEDOT fluorescein films after (a) wireless de-doping at 10 V for 10 minutes, conventional de-doping at (b) 0.0 V, (c) -0.2 V, and (d) -0.4 V, for 10 minutes in 0.1 M Li ClO_4. Arrows indicate the location of ImageJ analysis.
